# Cheminformatics Microservice: unifying access to open cheminformatics toolkits

**DOI:** 10.1186/s13321-023-00762-4

**Published:** 2023-10-16

**Authors:** Venkata Chandrasekhar, Nisha Sharma, Jonas Schaub, Christoph Steinbeck, Kohulan Rajan

**Affiliations:** Institute for Inorganic and Analytical Chemistry, Friedrich Schiller University Jena, Lessingstr. 8, 07743 Jena, Germany

**Keywords:** CDK, RDKit, Open Babel, Cheminformatics, Toolkits, Microservice

## Abstract

In recent years, cheminformatics has experienced significant advancements through the development of new open-source software tools based on various cheminformatics programming toolkits. However, adopting these toolkits presents challenges, including proper installation, setup, deployment, and compatibility management. In this work, we present the Cheminformatics Microservice. This open-source solution provides a unified interface for accessing commonly used functionalities of multiple cheminformatics toolkits, namely RDKit, Chemistry Development Kit (CDK), and Open Babel. In addition, more advanced functionalities like structure generation and Optical Chemical Structure Recognition (OCSR) are made available through the Cheminformatics Microservice based on pre-existing tools. The software service also enables developers to extend the functionalities easily and to seamlessly integrate them with existing workflows and applications. It is built on FastAPI and containerized using Docker, making it highly scalable. An instance of the microservice is publicly available at https://api.naturalproducts.net. The source code is publicly accessible on GitHub, accompanied by comprehensive documentation, version control, and continuous integration and deployment workflows. All resources can be found at the following link: https://github.com/Steinbeck-Lab/cheminformatics-microservice.

**Graphical Abstract:**

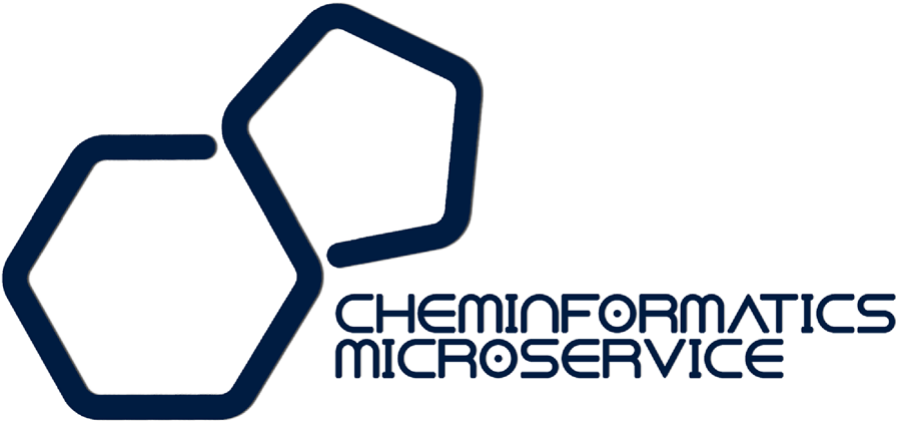

**Supplementary Information:**

The online version contains supplementary material available at 10.1186/s13321-023-00762-4.

## Introduction

Open cheminformatics toolkits, large-scale chemical databases, and an increase in available computing power have led to significant advancements in the field of cheminformatics in recent years [1, 2]. As a result, large amounts of chemical data can be handled and analysed efficiently, which in turn benefits research fields like chemistry, drug discovery, and material design. Multiple prominent open-source cheminformatics toolkits are available, which include RDKit [3], Open Babel [4], Chemistry Development Kit (CDK) [5, 6], Indigo [7], and the recently developed Python-based Informatics Kit for Analysing CHemical Units (PIKAChU) [8]. A summary of their native programming language, latest version, and licence information is given in Table 1.

**Table 1:** Summary of widely used open-source cheminformatics toolkits

Toolkit	Programming language	Latest version	Licence	Source code
Open Babel	C + +	3.1.1	GNU general public license (GPL)	https://github.com/openbabel/openbabel
CDK	Java	2.8.0	GNU lesser general public license v2.1	https://github.com/cdk/cdk
RDKit	C + + , Python	2023.03.2	BSD 3-clause license	https://github.com/rdkit/rdkit
PIKAChU	Python	1.0.13	MIT license	https://github.com/BTheDragonMaster/pikachu
Indigo	C/C + +	1.11.0	Apache license 2.0	https://github.com/epam/Indigo

All cheminformatics toolkits mentioned above offer ready-to-use routines for common tasks like data format conversions, descriptor calculations, and structure editing. On top of these, every toolkit has an individual set of more advanced functionalities like coordinate generation, substructure analysis, or structure normalisation. For this reason, researchers often use multiple toolkits in their tools or workflows [9, 10]. To do so, they have to familiarise themselves with the specific requirements, syntax, and algorithms of each employed toolkit. Being familiar with the underlying programming languages such as Python, Java, or C +  + is required to achieve this most efficiently. It is also essential to have a thorough understanding of chemical concepts, molecular representations, and computational algorithms.

For developing cheminformatics workflows, machine learning models, or web applications, researchers and software developers need to set up a proper working environment for the toolkits in order to integrate them into their applications. These software tools can later become cumbersome to use and maintain if not set up properly or inadequately documented. Unfortunately, this is often the case due to the complexity of the setup, lack of documentation, and lack of good research data management practices [11]. Other challenges in developing cheminformatics software, databases, and web applications result from building on top of these toolkits due to factors such as various toolkit version management [12], dependencies management [13], and maintenance [14].

- Software Version Management: The purpose of this process is to effectively manage the versions of software and toolkits throughout their development and maintenance cycle. The objective is to organise and track changes, facilitate collaboration, and ensure the stability and integrity of software projects, thus increasing productivity and streamlining development processes. This process can be time-consuming and requires careful planning and organisation.
- Dependencies: The majority of cheminformatics tools require several interdependent third-party libraries to function. Managing these dependencies can be challenging since developers must ensure that each dependency is installed correctly and compatible with the other dependencies. Otherwise, this can lead to conflicts between dependencies, which may cause the software to malfunction.
- Maintenance: It is challenging to maintain software and databases as they require regular updates and bug fixes to remain up-to-date and functional. In the special case of open-source software, this usually requires a large community of active and committed users and developers.

Most cheminformatics open-source programming toolkits have a rather high entrance barrier due to the abovementioned aspects. Also, the quality of available documentation and tutorials varies. This is especially critical for young researchers new to the field who have to spend a lot of setup time before being able to work on their research projects. Therefore, online tools for cheminformatics are becoming increasingly popular due to their usual ease of use, adequate documentation, and the fact that no or only little programming knowledge is required to use them [15–18]. The use of web-based solutions or solutions that are driven by Application Programming Interfaces (APIs) [19] offers a lower entrance barrier. In addition, these services are platform-independent and can be easily integrated with software utilities, databases, repositories, and cheminformatics data management workflows [17].

To overcome the challenging integration of third-party libraries like cheminformatics toolkits into application code, two common software development techniques can be used: containerization and microservices. Microservices [20], also referred to as microservice architecture, is a collection of small, autonomous services that can be deployed, scaled, and maintained individually [21]. By leveraging well-defined APIs, each microservice carries out a specific function and interacts with other microservices. These services are characterised by their granular nature and employ lightweight protocols to enable the independent execution of each service [22]. The native installation of such services on a system makes maintenance difficult in the long run. The service may not function as a result of Operating System (OS) level updates or software environment conflicts. In addition, in the event of a service failure, it is necessary to reboot the entire system. In order to address these issues, containerization can be used for the deployment of software services and applications. Containers are lightweight isolated environments that enable applications and their dependencies to run consistently across a variety of systems, independent of the OS [23]. In addition, a container provides a consistent and reproducible execution environment across development, testing, and production environments. In this work, containerization was achieved using Docker [24]. Software components can be containerized using Docker and distributed publicly via the Docker Hub [25], a cloud-based registry provided by Docker that allows developers to store, share, and distribute Docker images.

This article presents the Cheminformatics Microservice, an open-source solution for handling chemical data and performing various cheminformatics tasks by employing multiple cheminformatics toolkits (CDK, RDKit, and Open Babel). These tasks include generating high-quality chemical structure depictions and 3D conformers, calculating molecular descriptors and IUPAC names, and converting SMILES representations of chemical structures into other machine-readable formats. The microservice can be accessed through a unified REST (REpresentational State Transfer) [26] interface, which is made available as a public server for anyone to access via https://api.naturalproducts.net/. Alternatively, it can be installed locally or on a private server using the provided Docker image. It can also be deployed and auto-scaled on a Kubernetes-managed private cluster in just a few steps via the Helm Charts provided [27]. These deployment options are designed to be user-friendly since they require no prior knowledge of the underlying toolkits or their setup environment. They make the presented software service suitable for a wide range of applications in academic and industrial environments. The entire Cheminformatics Microservice source code is made available to the public on GitHub: https://github.com/Steinbeck-Lab/cheminformatics-microservice. Users and researchers are encouraged to submit feature requests and contribute to the microservice to ensure its continued growth.

## Implementation

**Fig. 1:**
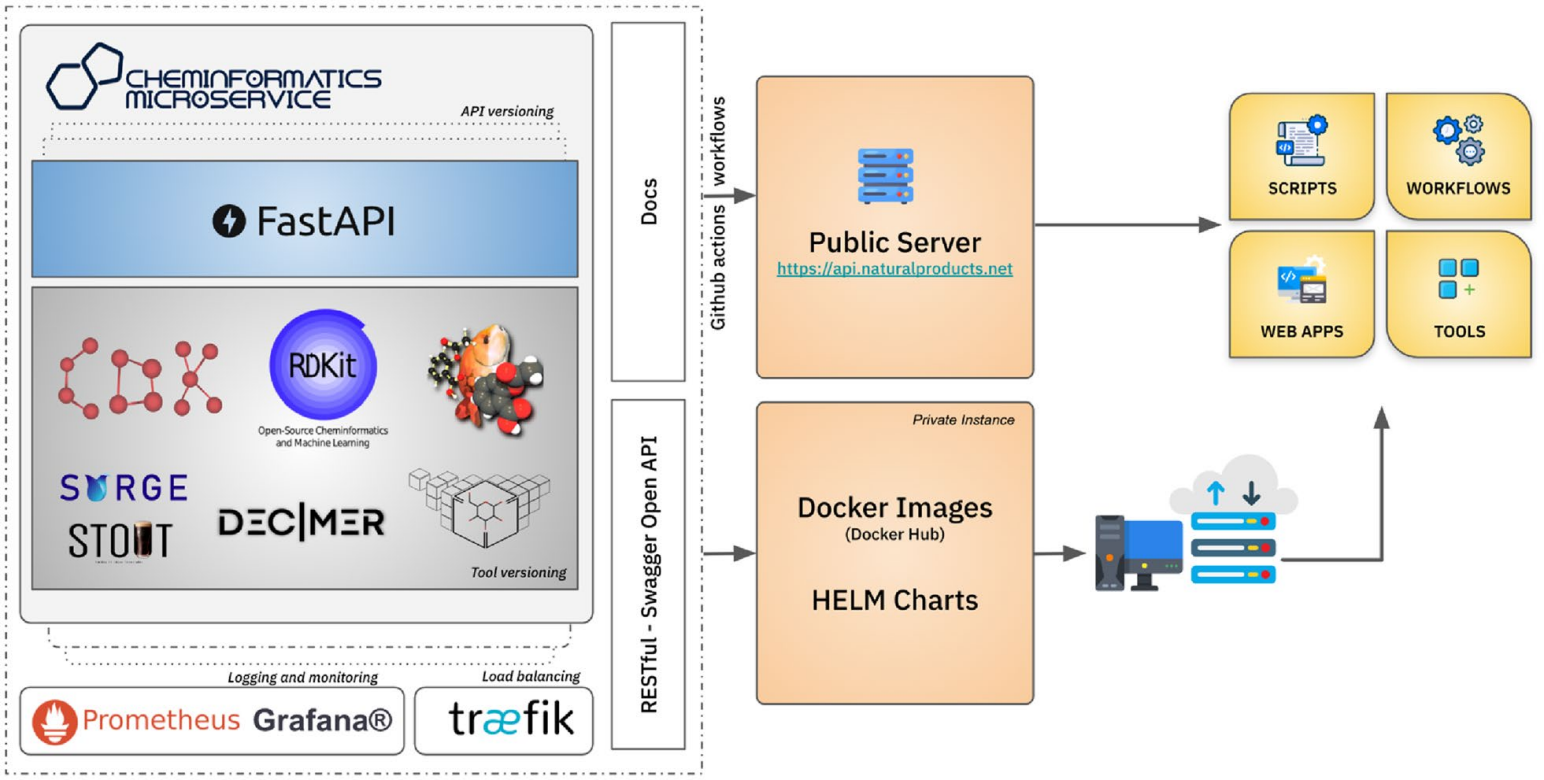
Overview of the Cheminformatics Microservice public server architecture. The public server can be accessed via https://api.naturalproducts.net/latest/docs

Cheminformatics Microservice is developed using FastAPI, a web framework for generating RESTful APIs with Python. FastAPI was chosen for this project due to its speed, efficiency, and suitability for building advanced APIs. It enables the straightforward creation of robust and scalable APIs. Docker is used for containerization, and semantic versioning principles are applied to track code changes and toolkit versions. In the microservice container, the cheminformatics toolkits RDKit and Open Babel are accessed natively using Python, while the Chemistry Development Kit (CDK) is integrated using JPype [28]. Cheminformatics Microservice consists of five modules, namely *chem*, *convert*, *depict*, *ocsr*, and *tools*. Compliant with the OpenAPI [29] specification version 3.1.0, this work provides standard documentation, encourages interoperability, enables automatic code generation, simplifies validation, and integrates with a variety of tools and libraries to enhance the functionality of REST (REpresentational State Transfer) APIs. An overview of the software architecture is given in Fig. 1.

The *chem* module offers various functions such as descriptor calculation, stereoisomer enumeration, HOSE code [30] generation, NPLikeness score calculation [31], ClassyFire classification [32], molecular structure standardisation [33], and a preprocessing pipeline for the upcoming version of the COCONUT database as explained in the results section below. The *convert* module provides conversions from SMILES [34] to other molecular string representations such as InChI [35, 36], InChIKey [36], canonical SMILES [37], CXSMILES [38], SELFIES [39, 40], and IUPAC names. The latter is achieved via the Smiles TO iUpac Translator (STOUT) version 2.0 toolkit [41]. Additionally, MOL files can be generated with 2D and 3D coordinates from SMILES input. Using the *depict* module, one can generate 2D depictions of chemical structures with various settings, including an option to generate 2D representations with stereochemical annotations following the Cahn–Ingold–Prelog [42] (CIP) sequence rules. RDKit or CDK can be used to generate 2D representations. The depictions are generated in an SVG format and may be scaled to fit the needs of the user. It is also possible to generate a 3D depiction [43] using this module, which is useful as a chemical structure display option for databases or as a teaching aid. The underlying 3D conformer is generated using RDKit. The *ocsr* module incorporates Deep lEarning for Chemical ImagE Recognition (DECIMER) modules [44, 45] for translating images of chemical structures into machine-readable SMILES representations. These can be accessed via HTTP *POST* requests. Finally, the *tools* module is designated as a miscellaneous collection of advanced cheminformatics tools. It offers a function to generate chemical structures from a molecular formula given as input using the surge chemical graph generator [46]. Other functions of the *tools* module can be used to identify glycosidic moieties in input molecules and to remove them in order to generate the aglycone structure. These routines are implemented based on the Sugar Removal Utility (SRU) [47].

The functionalities offered by each main module described above can also be implemented via independent Python functions, enabling users to access Cheminformatics Microservice natively without the REST interface. Users can import and use it like any other package directly in their own Python code. Individual toolkit wrapper modules provide access to the three cheminformatics toolkits RDKit, CDK, and Open Babel. RDKit and Open Babel are natively accessible, while CDK, a Java library, has functionalities ported to Python using JPype. It is possible to extend the functionalities provided by cheminformatics toolkits in the future by using these wrapper modules. Separating them into individual modules is necessary to achieve granular control over the functions. This also ensures that the entire system will not be broken if one module is affected by potential software failures. The Python functions are documented separately, and the documentation can be accessed via: https://cheminformatics-microservice.readthedocs.io/en/latest/.

To ensure reproducibility, a consistent versioning system is essential. Best practices for research data management [48, 49] recommend documenting software and component versions separately, especially for tools like the presented microservice with multiple dependencies. Cheminformatics Microservice uses multi-level versioning to record API and software dependencies. The codebase undergoes bi-annual major releases, with corresponding documentation provided for the underlying toolkits, tools, and environment dependencies for each release. The API version updates are released only when significant changes to the API endpoints have been made. It is possible to update the underlying cheminformatics toolkits whenever new releases are published without having to update the entire code base since the REST API remains unchanged. The API usage can be logged, monitored, and visualised using Prometheus [50] and Grafana [51] in a standalone or distributed environment. Cheminformatics Microservice can also be deployed using a Continuous Integration and Continuous Deployment (CI/CD) pipeline via GitHub Actions [52]. Code integration, testing, and application deployment are automated using CI/CD, which fosters collaboration, minimises manual tasks, and enables timely feedback.

## Results and discussion

The presented Cheminformatics Microservice provides straightforward access to the open-source cheminformatics toolkits RDKit, CDK, and OpenBabel, as well as deep learning-based tools such as Deep lEarning for Chemical ImagE Recognition (DECIMER) for OCSR and Smiles TO iUpac Translator (STOUT). Building on top of these tools and toolkits, it makes a diverse selection of important functionalities needed by cheminformaticians on a daily basis accessible via a RESTful interface. Additionally, Cheminformatics Microservice includes a number of widely used packages, like the ChEMBL [53] curation pipeline [33], CDK-based sugar removal functionalities [47], and the open-source structure generator surge [46]. The presented microservice is intended to facilitate the handling of large amounts of chemical structural data to enable the development of adaptable, scalable, and maintainable cheminformatics applications.

**Fig. 2:**
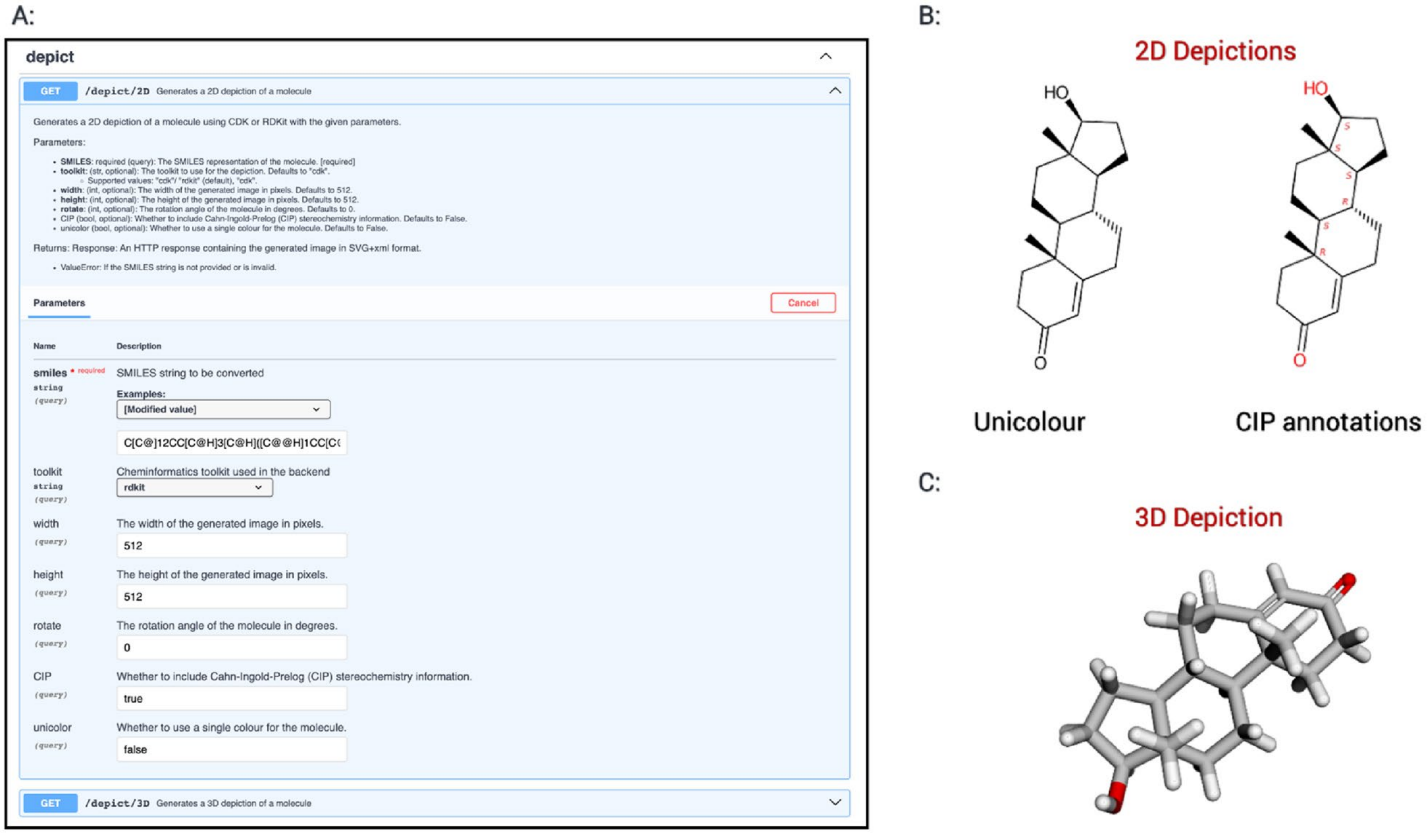
**A** The screenshot showcases the output of the module, displaying the depiction options for the input molecule Testosterone given as a SMILES string. **B** The output of the *depict* module with unicolor and CIP annotations for 2D depictions, and **C** the interactive 3D depiction [59]

The main modules of Cheminformatics Microservice are well-documented and can be accessed via the following link: https://api.naturalproducts.net/. In order to obtain the output generated by the microservice, each API module uses either a *GET* or a *POST* HTTP request method [54]. Most of the services provided by the *chem, convert,* and *depict* modules can be accessed using SMILES as an input format for molecular structures. Where a specific functionality provided by the microservice can be achieved in a similar manner with RDKit, CDK, or Open Babel, the user has the option of employing the toolkit of their choice via an additional parameter. The *chem* module offers routines for structure manipulation and standardisation, descriptor calculation, and chemical classification. The *standardize* functionality is used via a *POST* method for the purpose of standardising molecules represented by MOL format tables through the ChEMBL structure curation pipeline. The *convert* module enables users to convert SMILES representations into other formats of their choice, using the *GET* method. Meanwhile, the *depict* module allows users to generate customised 2D depictions of molecular structures, offering options for coloured or black and white output images. The stereochemical annotations on the 2D depictions are generated using the Cahn-Ingold-Prelog (CIP) priority rules. To accomplish this, the Java package *centres* [55, 56] is used in conjunction with CDK. Additionally, the *depict* module is able to produce interactive 3D models of input structures. Figure 2A, B illustrates the application of the depict module to generate a 2D depiction with CIP annotations in colour on a scale of 512 × 512 pixels and with a 52° rotation. The depicted image with CIP annota*depict*tions can be generated directly by using this call to the API: https://api.naturalproducts.net/latest/depict/2D?smiles=C%5BC@%5D12CC%5BC@H%5D3%5BC@H%5D(%5BC@@H%5D1CC%5BC@@H%5D2O)CCC4=CC(=O)CC%5BC@%5D34C&width=512&height=512&rotate=52&&CIP=true&unicolor=false&toolkit=cdk

2D representations are produced in the form of SVG images, whereas 3D representations are returned as HTML files containing embedded JSMol objects [57, 58].

Through REST API calls, the *tools* and *ocsr* modules offer convenient access to the underlying functionalities of the integrated tools. For example, the *tools* module allows users to employ the open-source chemical structure generator surge to generate chemically valid structures based on a provided molecular formula. The generated structures are returned as a list of SMILES representations. In order to address the resource-intensive nature of the chemical structure generation process, a maximum limit of 10 heavy atoms in the given molecular formula has been imposed. This restriction applies exclusively to the public instance. The *tools* module also allows users to access the Sugar Removal Utility (SRU) to detect and remove linear and circular sugar moieties in/from input structures. Users can access the DECIMER toolkit through the *ocsr* module, which enables identification, segmentation, and translation into machine-readable representations of chemical structure depictions from the scientific literature.

Currently, Cheminformatics Microservice is available to the public via https://api.naturalproducts.net/latest/docs and in the back end, the container is running on a compute server with the processor Intel(R) Xeon(R) Gold 6226R CPU @ 2.90 GHz and 16 GB of RAM.

### Use of Cheminformatics Microservice in the COCONUT database

**Fig. 3:**
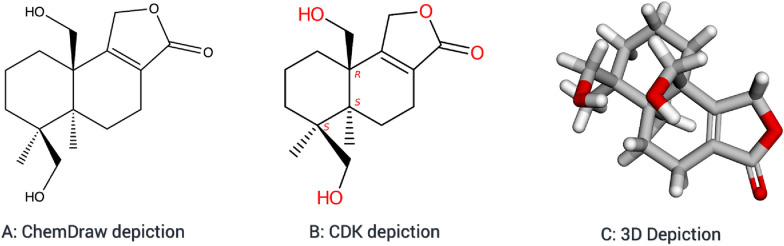
The molecule Astellolide W [60] was extracted from the referenced article using DECIMER. The structure is depicted using ChemDraw (**A**) and CDK (**B**) for 2D representations, while the 3D depiction (**C**) was created using the presented microservice

Cheminformatics Microservice is extensively employed in the upcoming version of the COCONUT (COlleCtion of Open Natural prodUcTs) database that is currently under development. With the *depict* module, it becomes feasible to present all the natural product structure data entries within COCONUT in both 2D and 3D formats (Fig. 3). The microservice also includes the generation of molecular descriptors and further preprocessing steps for data submission to the COCONUT database.

### Documentation

**Fig. 4:**
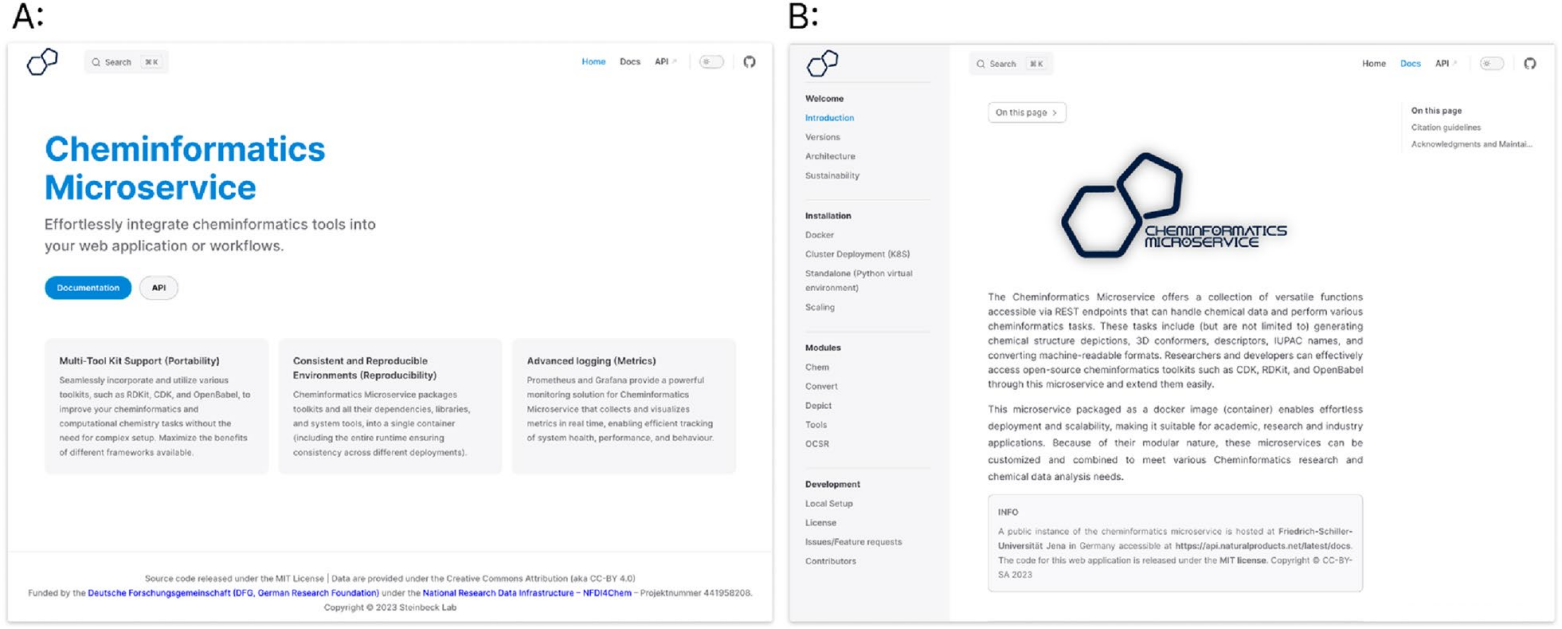
Screenshots of the Cheminformatics Microservice landing page (**A**) and detailed documentation (**B**)

Cheminformatics Microservice offers detailed documentation alongside its source code, ensuring that users can easily access and navigate it without a high entry barrier. The documentation provides clear guidance on how to effectively use, deploy, and install the software. Figure 4 offers a glimpse of the documentation that is deployed using GitHub Pages and can be accessed at the following URL:

https://docs.api.naturalproducts.net.

### Performance and scalability

Scaling is particularly important for those who plan to use the API endpoints for database generation, large-scale descriptor calculation, or automated literature mining since it considerably reduces the overall computation time required. Cheminformatics Microservice is specifically designed to scale at large, in a local deployment as well as an orchestrated cluster. It can be configured to run on multiple workers when deployed independently. If deployed through Docker Compose or over a Kubernetes cluster, the microservice can be auto-scaled infinitely to handle incoming requests. Helm Chart or the Docker Compose file provided in the codebase enable scaling without any additional setup. Prometheus and Grafana are used to monitor the request count over time. These logs and other performance indicators will facilitate the scaling of Cheminformatics Microservice workers based on user demand in the future.

**Fig. 5:**
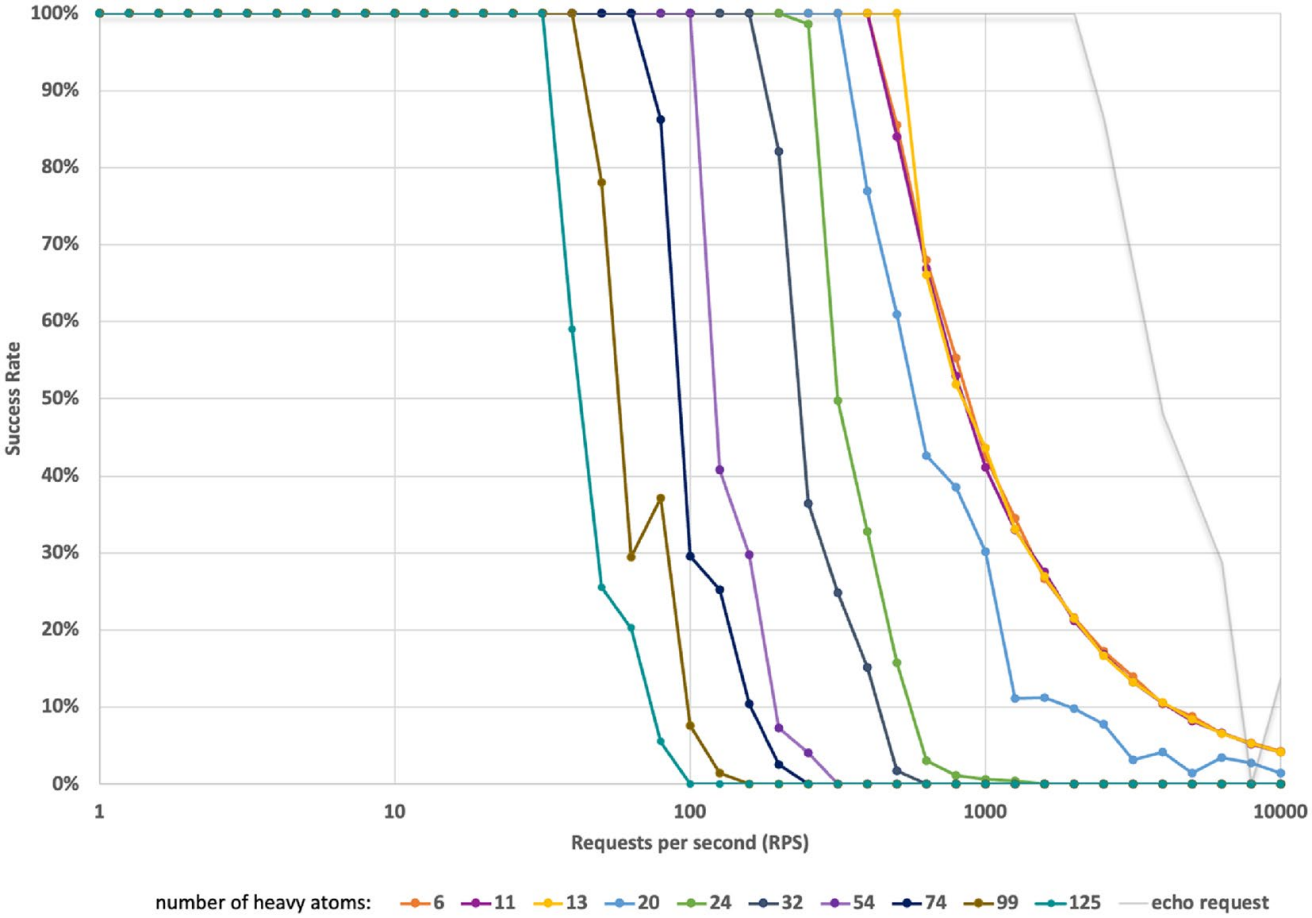
A snapshot of performance comparing the success rate with an increase in the number of requests per second as well as with an increase in the number of heavy atoms in the input. The grey line represents the maximum number of requests the server could process (echo request)

To determine scalability, stress testing was performed using Vegeta [61]. To determine the maximum throughput Cheminformatics Microservice can handle when deployed on a machine with Intel(R) Xeon(R) Gold 6226R CPU @ 2.90 GHz and 16 GB of RAM, requests in small increments were added using Vegeta and the delivered throughput was measured until a limit was reached (Fig. 5). This stress test indicated that the service reached its maximum capacity when handling approximately 2500 requests per second for echo requests, after which the success rate began to decline. When queried with the task to generate 2D coordinates for a molecular structure given as a SMILES string using RDKit, Fig. 5 clearly demonstrates that the microservice can effectively handle a wide range of requests, ranging from 50 to 500 per second. With an increase in the input molecule size and number of requests per second, this rate starts to deteriorate. A detailed description of the stress test can be found in the Additional file 1.

Nevertheless, the default configuration of Cheminformatics Microservice ensures excellent stability in handling user requests and effectively manages to execute the computation required.

However, limitations are imposed on the public instance for some specific tools and routines. For example, restrictions are imposed in the structure generator surge when dealing with molecules containing more than ten heavy atoms, as this service demands significant computational resources. Access to mass mining through the DECIMER endpoint is also restricted. These restrictions might be reconsidered in the future, depending on the user demand and computation resource availability.

## Conclusion

The presented Cheminformatics Microservice is a self-contained, web-based service that operates independently of the operating system. This can be used by researchers with no or limited programming experience to perform daily cheminformatics tasks effortlessly via the API hosted at https://api.naturalproducts.net. This service allows a diverse range of open-source cheminformatics toolkits to be accessed without requiring any software installation or environment setups. To the best of our knowledge, it is currently the sole microservice in the field of cheminformatics offering users the ability to access multiple toolkits, as well as additional tools such as the open-source structure generator surge, Sugar Removal Utility (SRU), and the DECIMER OCSR tools. Cheminformatics Microservice is designed to be user-friendly, easily extendable, deployable, and scalable. It can be accessed through the public API or hosted on private clusters or single machines.

The integration of multiple cheminformatics toolkits in a centralised platform enhances user accessibility to cheminformatics utilities. By using industry-standard monitoring, deployment, documentation, and code quality standards, this project demonstrates software development in a user-focused manner. By making the source code and the documentation completely open and public, we aim to make valuable contributions to the scientific community while also enabling the submission of feature requests for future enhancements. Cheminformatics Microservice is anticipated to become an invaluable asset for cheminformaticians due to its inherent expandability. It follows a clear semantic versioning system, receives bi-annual updates, and includes comprehensive documentation. These characteristics significantly facilitate data reproduction and reuse for researchers, thereby fostering better collaboration among peers.

## Availability and requirements

- Project name: Cheminformatics Microservice
- Project home page: https://github.com/Steinbeck-Lab/cheminformatics-microservice
- Docker Image: https://hub.docker.com/r/nfdi4chem/cheminformatics-microservice
- Helm Chart repo: https://nfdi4chem.github.io/repo-helm-charts/
- Helm Chart GitHub: https://github.com/NFDI4Chem/repo-helm-charts
- Current version: v1.6.0
- DOI of archived current release: https://doi.org/10.5281/zenodo.7745987
- Operating system(s): Independent
- Programming language: Python 3, HTML
- Requirements:
  - API calls:
    - Internet connection and command line interface or a web browser
  - Run locally:
    - Docker—To use Cheminformatics Microservice as a Docker container.
    - Conda environment—to use Cheminformatics Microservice natively without Docker.
  - Dependencies (managed by Docker/Conda):
    - Python packages: uvicorn ≥ 0.15.0, < 0.16.0, fastapi ≥ 0.80.0, fastapi-pagination =  = 0.10.0, fastapi-versioning ≥ 0.10.0, prometheus-fastapi-instrumentator, jpype1 =  = 1.4.1, jinja2, pandas, chembl_structure_pipeline, HOSE_code_generator @ git + https://github.com/Ratsemaat/HOSE_code_generator, websockets =  = 10.4, pillow =  = 9.4.0, opencv-python =  = 4.7.0.68, matplotlib =  = 3.4.3, scikit-image, pdf2image =  = 1.16.2, IPython, pystow **≥** 0.4.9, unicodedata2 =  = 15.0.0, efficientnet, tensorflow =  = 2.12.0, pillow-heif =  = 0.10.0, selfies ≥ 2.1.1, httpx ≥ 0.24.1, keras_preprocessing =  = 1.1.2, decimer-segmentation ≥ 1.1.2, STOUT-pypi ≥ 2.0.5 and decimer ≥ 2.2.0
    - Java: OpenJDK for Java 11
    - Java Libraries: CDK 2.8.0, SRU 1.3.2 and Centres 1.0
- Licence: MIT
- Documentation:
  - Home page: https://docs.api.naturalproducts.net/
  - API: https://api.naturalproducts.net/latest/docs
  - Python Documentation: https://cheminformatics-microservice.readthedocs.io/en/latest/
- Any restrictions to use by non-academics: None.

### Supplementary Information

**Additional file 1: Performance/Stress test results.**

## Data Availability

Data availability Not Applicable.

## Abbreviations

2D: Two dimensional
3D: Three dimensional
APIs: Application Programming Interfaces
BSD: Berkeley Software Distribution
CC: Creative Commons
CDK: Chemistry Development Kit
CI/CD: Continuous Integration and Continuous Deployment
CIP: Cahn–Ingold–Prelog
COCONUT: COlleCtion of Open Natural prodUcTs
CPU: Central processing unit
CXSMILES: Chemaxon Extended Simplified Molecular Input Line Entry Specification
DECIMER: Deep lEarning for Chemical ImagE Recognition
GB: Gigabyte
GHz: Gigahertz
GNU: GNU’s Not Unix
GPL: General Public License
HOSE: Hierarchically ordered spherical environment
HTTP: Hypertext Transfer Protocol
HTML: Hypertext Markup Language
InChI: International Chemical Identifier
IUPAC: International Union of Pure and Applied Chemistry
JDK: Java Development Kit
JPype: Java for Python
JSMol: JavaScript molecular viewer
MIT: Massachusetts Institute of Technology
NPLikeness: Natural product likeness
OCSR: Optical chemical structure recognition
OS: Operating system
PC: Personal computer
PIKAChU: Python-based Informatics Kit for Analysing CHemical Units
RAM: Random access memory
REST: REpresentational state transfer
SELFIES: SELF-referencIng embedded strings
SMILES: Simplified molecular input line entry specification
SRU: Sugar Removal Utility
STOUT: SMILES-TO-IUPAC-name translator
SVG: Scalable Vector Graphics
