# Supplementary material for: Cheminformatics Microservice: unifying access to open cheminformatics toolkits
Source: J Cheminform. 2023 Oct 16;15:98. doi: 10.1186/s13321-023-00762-4 (PMC10577930; doi:10.1186/s13321-023-00762-4)

# Additional file Information

**Performance testing:** Latency distribution and success rate at each request rate

**Ramping Load:** To determine the maximum throughput the Cheminformatics Microservice public instance (Intel(R) Xeon(R) Gold 6226R CPU @ 2.90GHz and 16GB of RAM) can handle, we used the Vegeta (<https://github.com/tsenart/vegeta>) tool to add load in small increments and measure the delivered throughput until a limit was reached. The results were then graphed to show the scalability profile. The microservice’s ***create 2D coordinates from SMILES*** endpoint was chosen as the test request, and the scalability profiles were generated for requests with input structures submitted as SMILES representations starting from 6 heavy atoms to 125 heavy atoms (randomly sampled from the COCONUT database). The scalability profiles were then inspected visually to assess the server performance (SI Figure A - K).

| SMILES | Coconut ID | Heavy atom count |
| --- | --- | --- |
| Echo request | - | - |
| O=C(N)C(F)Cl | CNP0031243 | 6 |
| OC1CC2N(C)C(C1)C(O)C2 | CNP0205916 | 11 |
| O=C1C=C(O)C(C(=O)C1(C)C)(C)C | CNP0103604 | 13 |
| OOC(C=CC#CC#CC(O)C=C)CCCCCCC | CNP0171228 | 20 |
| O=C1NC(C)C2C(NC(=O)N(C)C2C3=CC=C(C=C3)[NH+]([O-])O)N1C | CNP0469758 | 24 |
| O=C(C1=C(O)C=C(OC)C=C1OC2OC(CO)C(O)C(O)C2O)CCC3=CC=C(O)C=C3 | CNP0267058 | 32 |
| O=C1OC(C)CCCC(=O)CCCC=CC=2C=C(O)C(=C(O)C12)C(C3=CC(OC)=C(OC)C(OC)=C3)CC(=O)NC(C4=NC=5C=CC=CC5N4)C(C)CC | CNP0319430 | 54 |
| O=C(O)C1OC(OC2CCC3(C)C(CCC4(C)C3CC=C5C6CC(C)(C)CCC6(C(=O)OC7OCC(O)C(O)C7OC8OC(C)C(OC9OCC(O)C(O)C9O)C(O)C8O)C(O)CC54C)C2(C)C)C(O)C(O)C1O | CNP0187011 | 74 |
| O=C(O)CCC(NC(=O)C(NC(=O)C=1N=C(SC1)C(=O)C(C)CC)CC(C)C)C(=O)NC(C(=O)NCCCCC2NC(=O)C(NC(=O)C(NC(=O)C(NC(=O)C(NC(=O)C(NC(=O)C(NC2=O)CCCN)C(C)CC)CC=3C=CC=CC3)CC4=CN=CN4)CC(=O)O)CC(=O)N)C(C)C | CNP0170666 | 99 |
| O=C(O)C1(OC2C(O)C(OC(CO)C2O)OC3C(O)C(OC(OC4C(OC(OC5C(O)C(O)C(OCC(NC(=O)CCCCCCCCCCCCCCC)C(O)CCCCCCCCCCCCCCC)OC5CO)C(O)C4OC6(OC(C(O)C(O)CO)C(NC(=O)C)C(O)C6)C(=O)O)CO)C3CC(=O)C)CO)OC(C(O)C(O)CO)C(NC(=O)C)C(O)C1 | CNP0001893 | 125 |

*Note: In the following plots, all axes are logarithmic. The complete performance results are available on Zenodo -* [*https://zenodo.org/record/8124294*](https://zenodo.org/record/8124294)

1. Scalability profile - Echo request


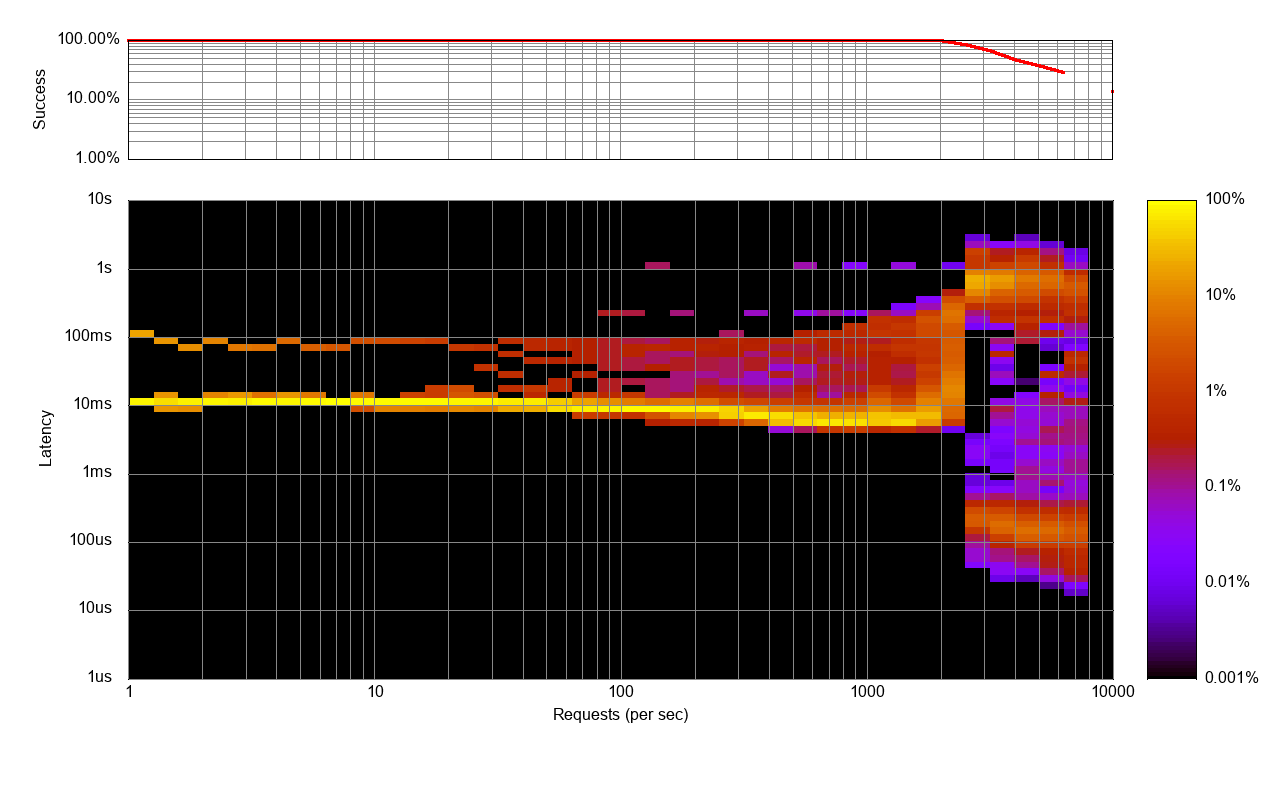


1. Scalability profile - SMILES to Mol2D Conversion (6 heavy atoms)


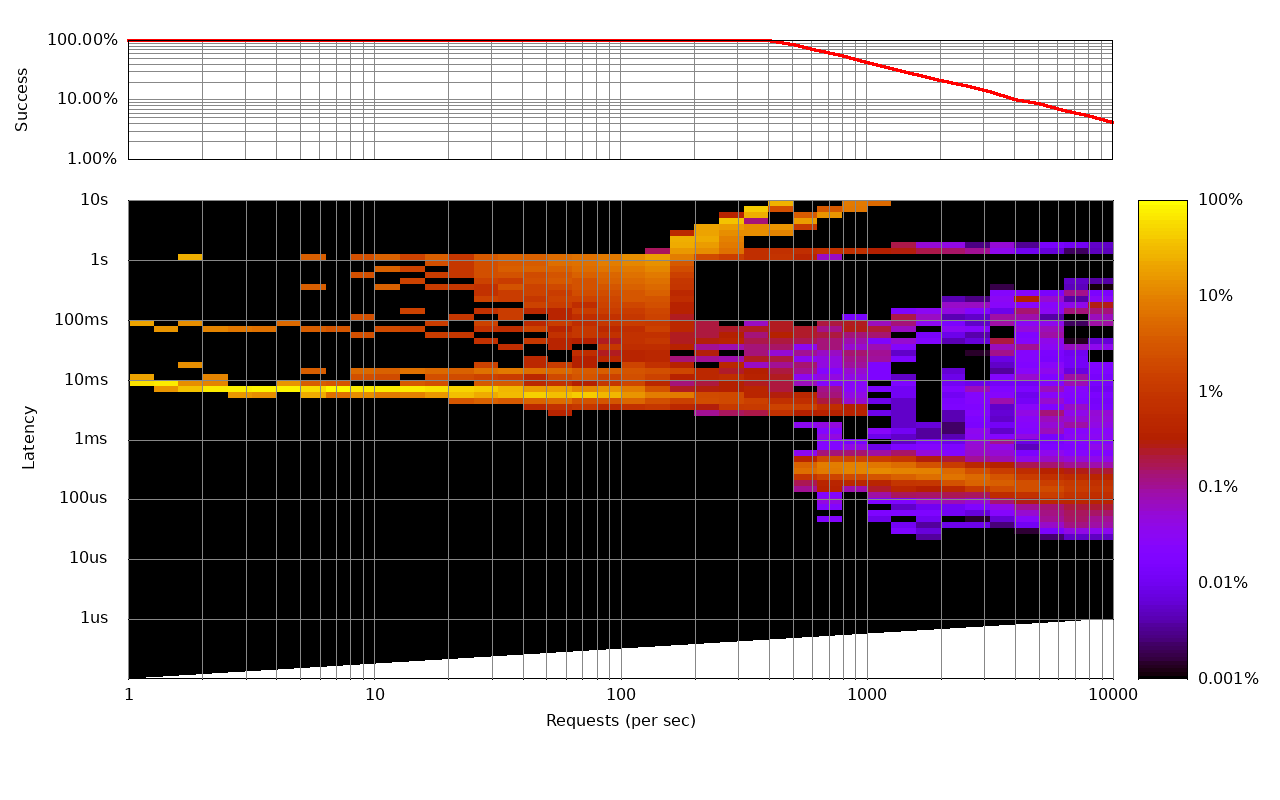


1. Scalability profile - SMILES to Mol2D Conversion (11 heavy atoms)


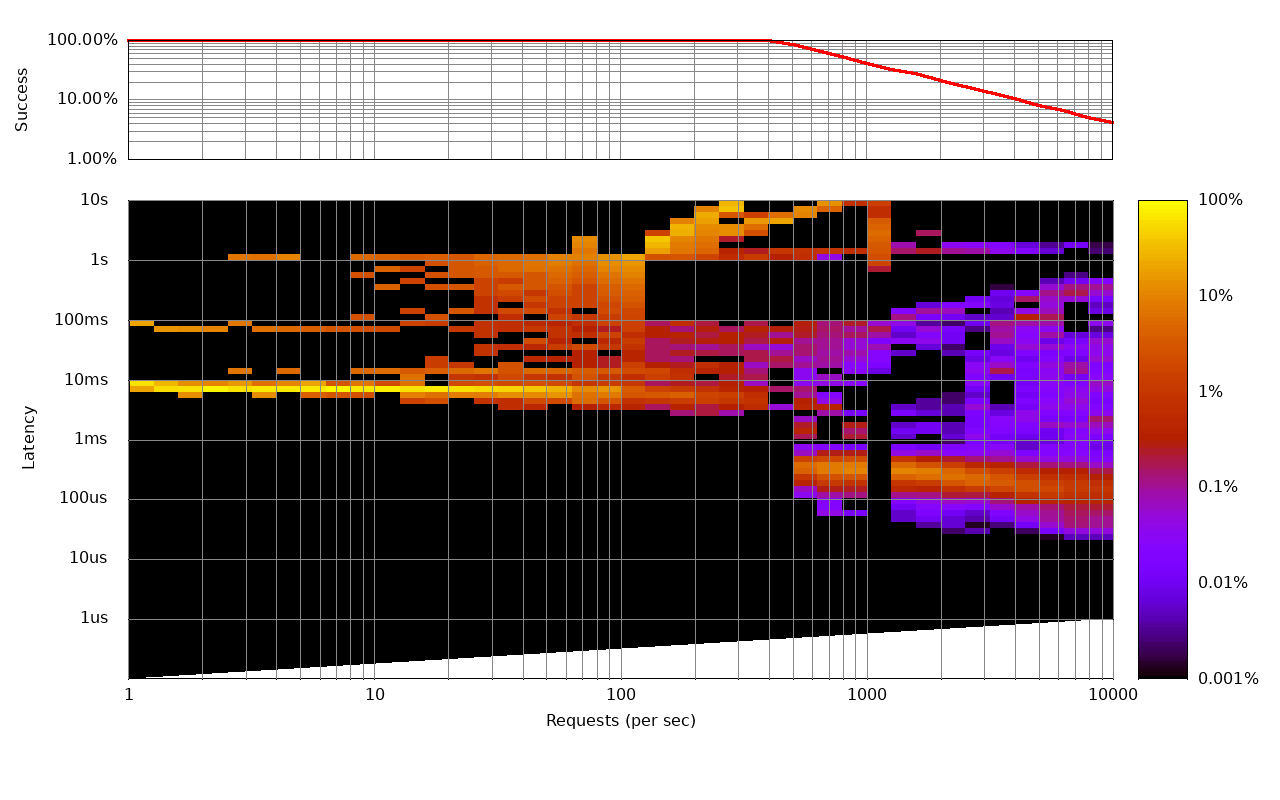


1. Scalability profile - SMILES to Mol2D Conversion (13 heavy atoms)


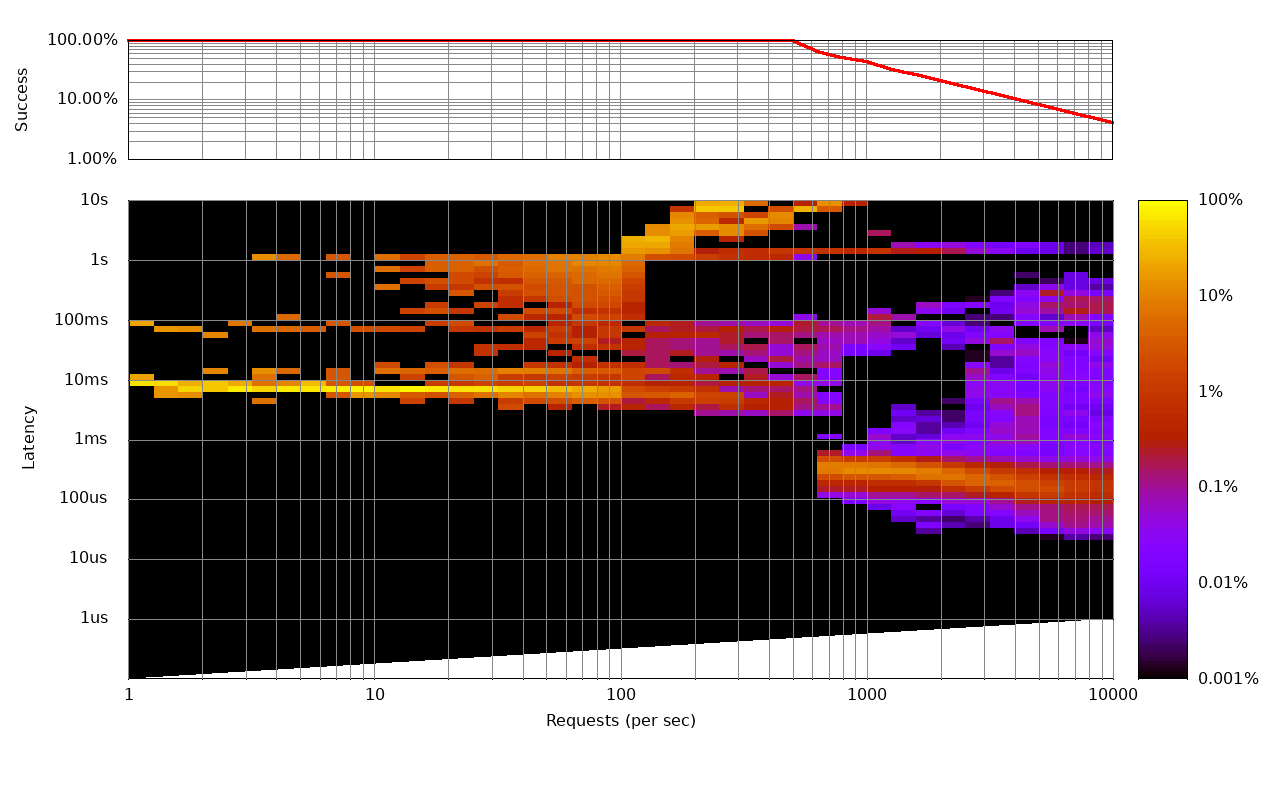


1. Scalability profile - SMILES to Mol2D Conversion (20 heavy atoms)


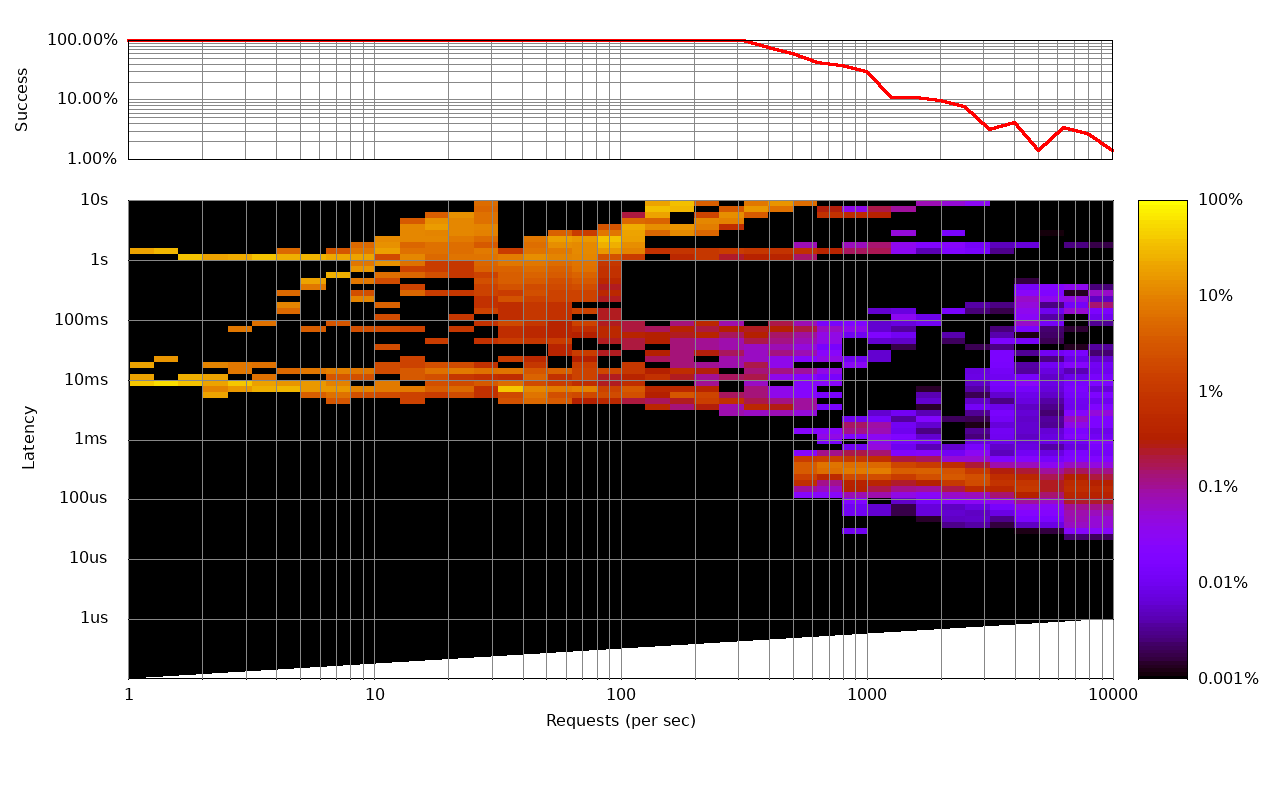


1. Scalability profile - SMILES to Mol2D Conversion (24 heavy atoms)


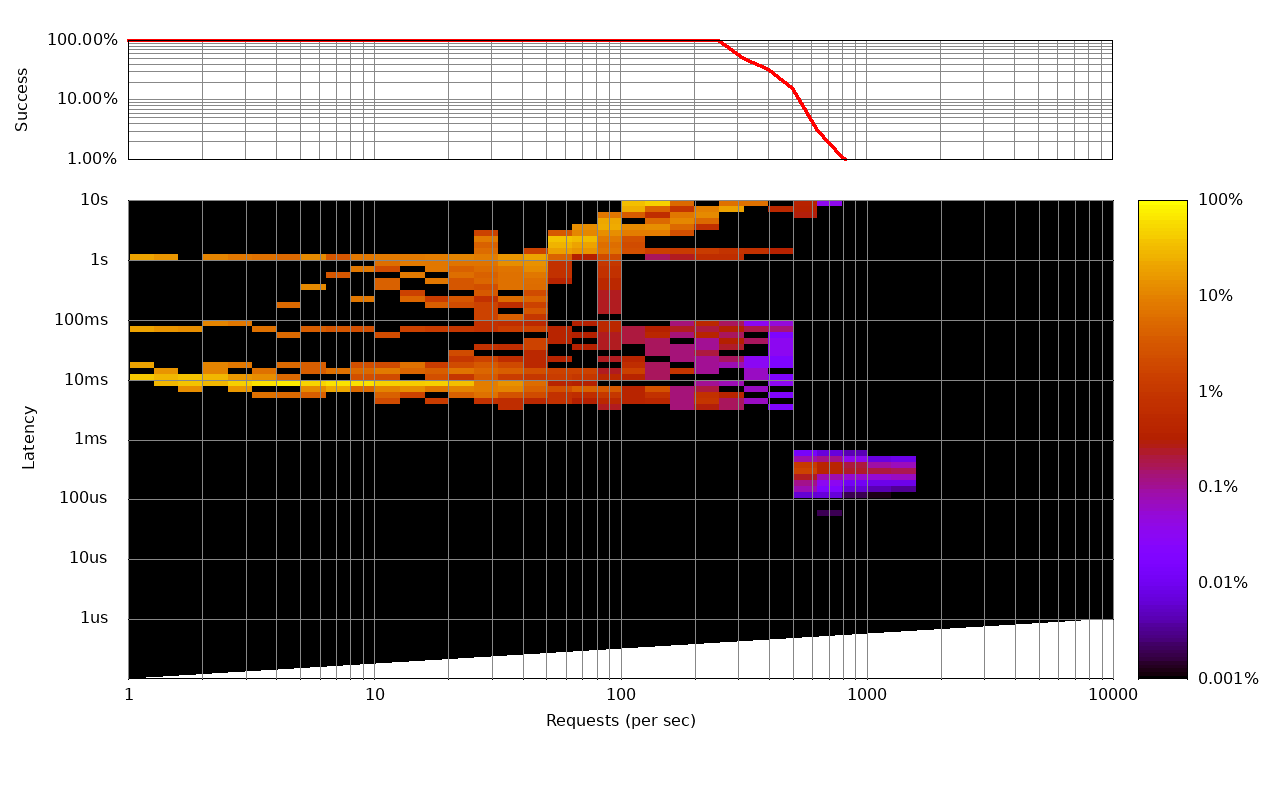


1. Scalability profile - SMILES to Mol2D Conversion (32 heavy atoms)


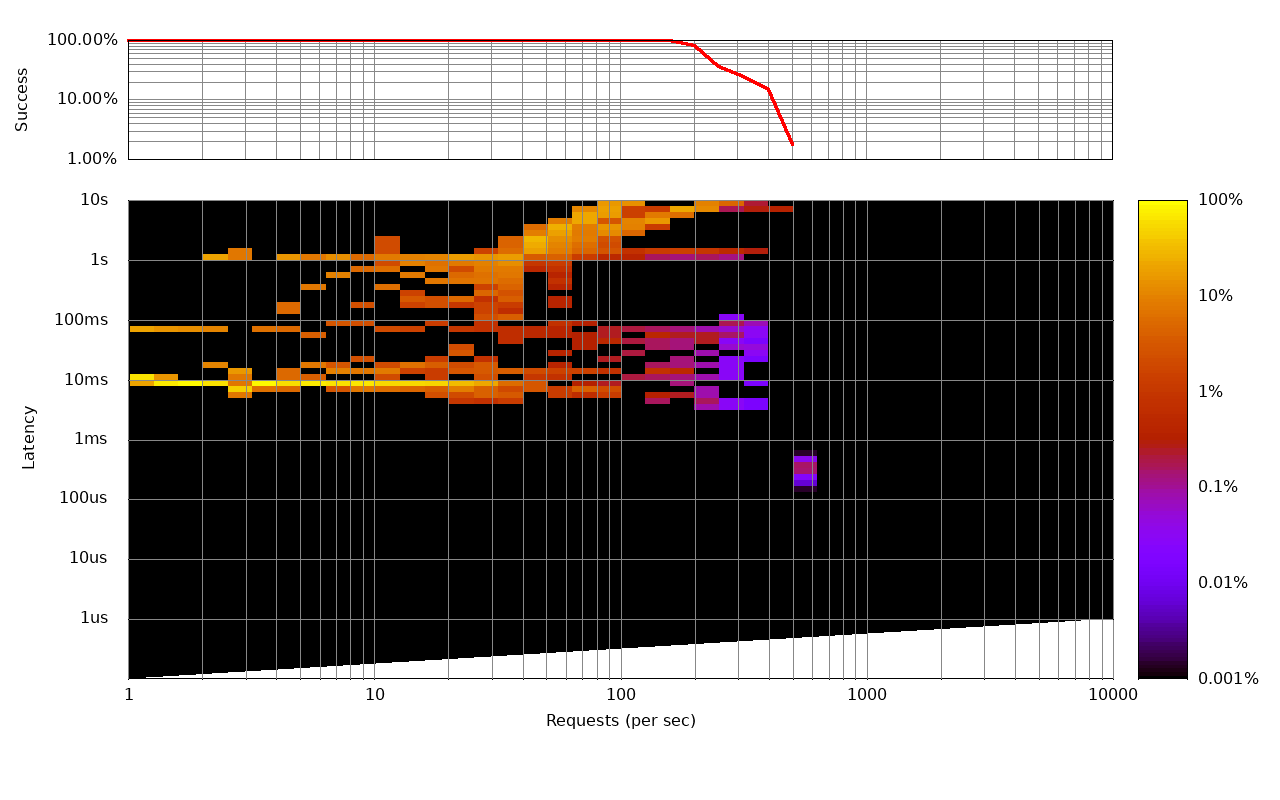


1. Scalability profile - SMILES to Mol2D Conversion (54 heavy atoms)


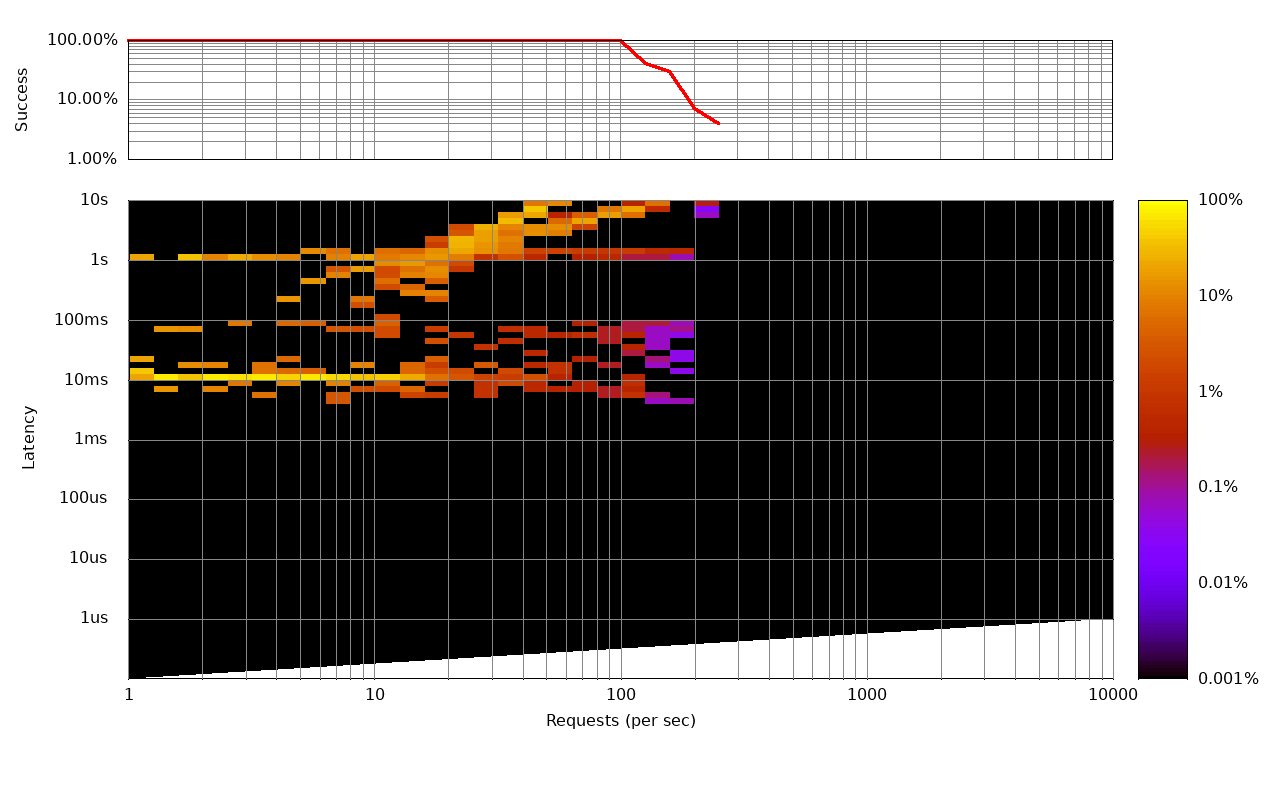


1. Scalability profile - SMILES to Mol2D Conversion (74 heavy atoms)


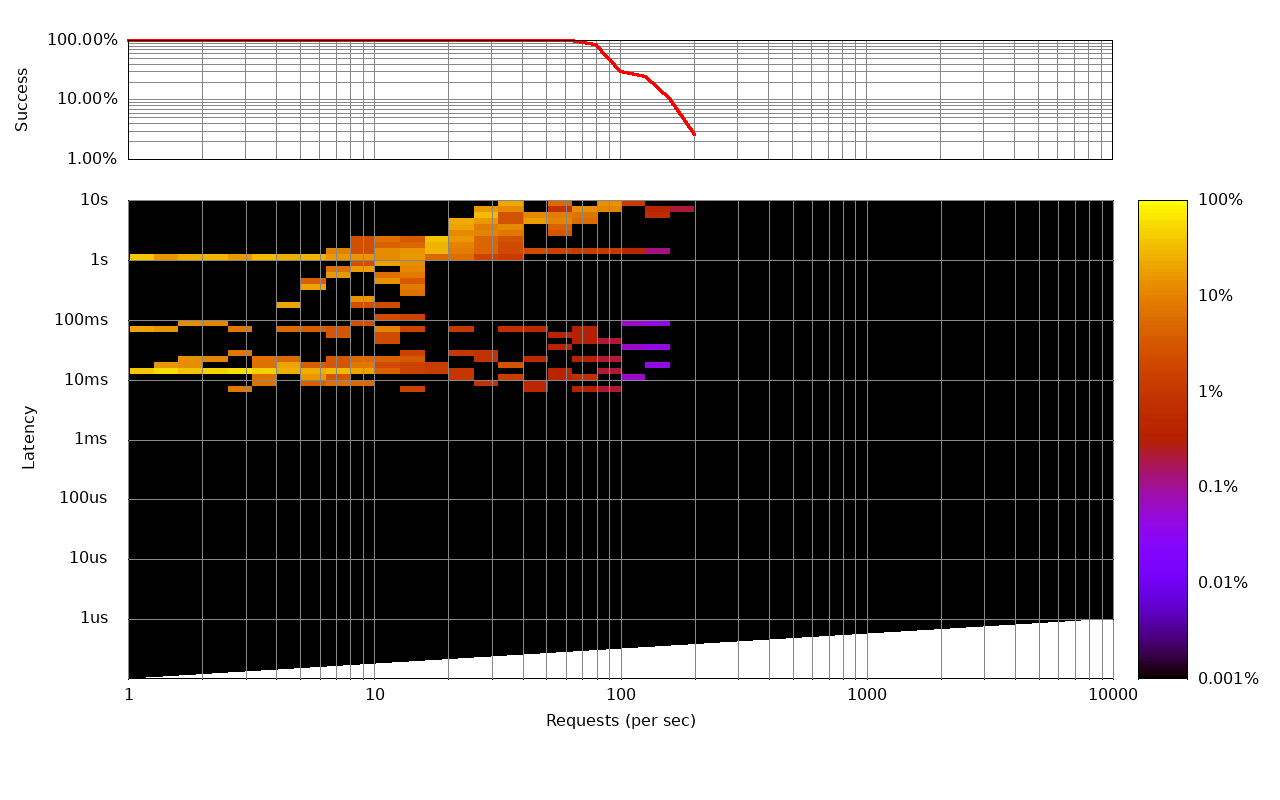


1. Scalability profile - SMILES to Mol2D Conversion (99 heavy atoms)


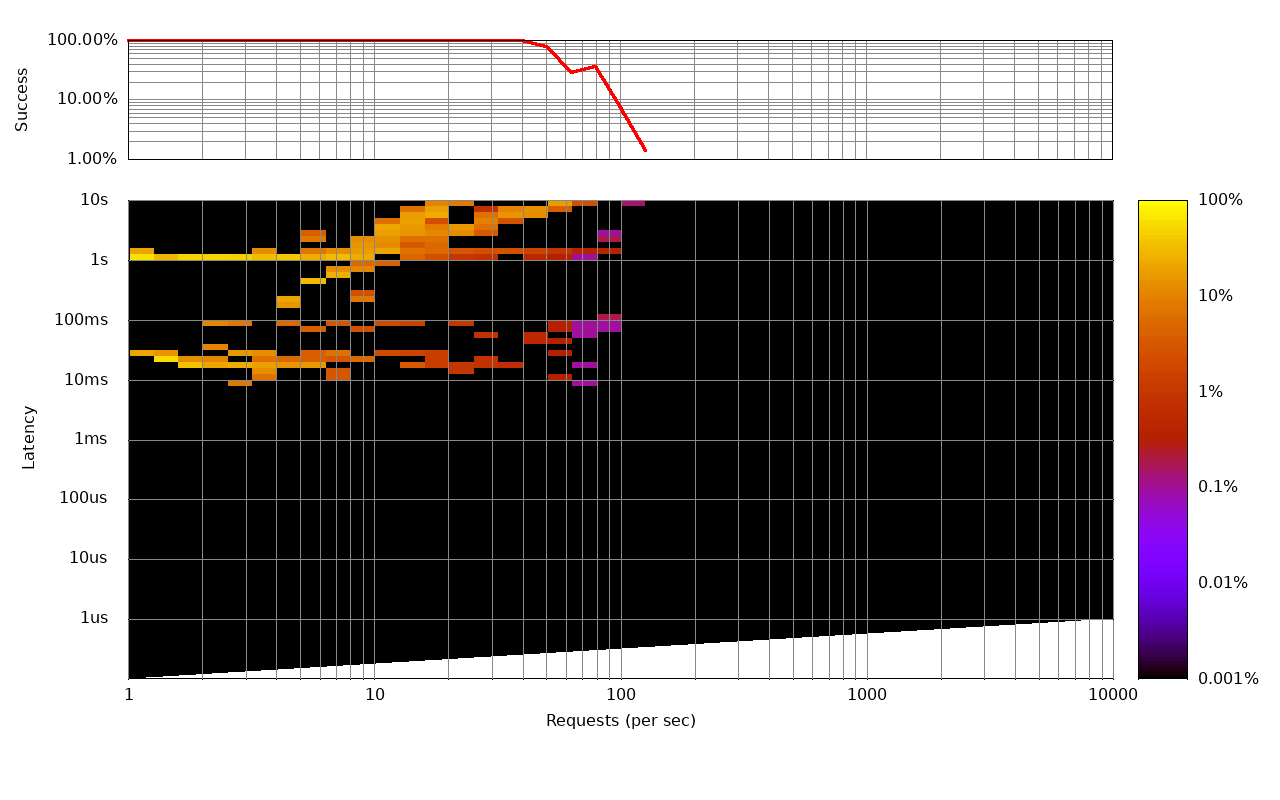


1. Scalability profile - SMILES to Mol2D Conversion (125 heavy atoms)


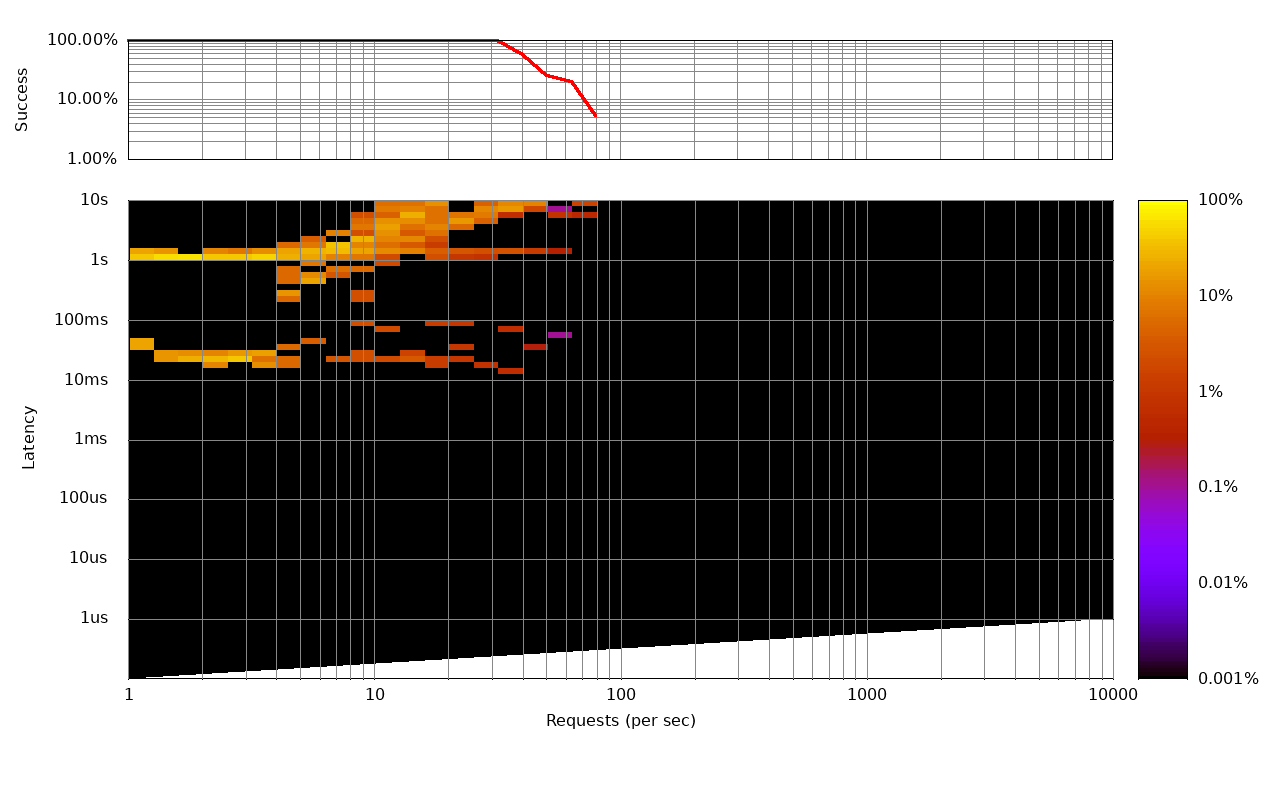

Supplement: Supplementary file 1 — Additional file 1: Performance/Stress test results. [file 13321_2023_762_MOESM1_ESM.docx]
